# Supplementary material for: Patterns of Online Stress Management Information-Seeking Behavior in Hungary
Source: Int J Environ Res Public Health. 2025 Mar 22;22(4):473. doi: 10.3390/ijerph22040473 (PMC12027446; doi:10.3390/ijerph22040473)
Supplement: Supplementary file 1 [file ijerph-22-00473-s001.zip › ijerph-3411297-supplementary.pdf]

ISSP 2021 – Health and Health Care II
Source Questionnaire

(November 2020)

Bibliographic citation:

ISSP Research Group (2021): International Social Survey Programme: ISSP 2021 – Health and Health Care II: Source questionnaire.

International Social Survey Programme

2021 Health and Health Care II

Final source questionnaire*

November 30, 2020

Health and Health Care II

Drafting group:
Iceland, Convener (Sigrún Ólafsdóttir, Guðbjörg Andrea Jónsdóttir)
Czech Republic (Dana Hamplova, Jindrich Krejci)
India (Yashwant Deshmukh)
Israel (Noah Lewin-Epstein)
South Africa, (Jarè Struwig, Benjamin Roberts)

Additional guest member:
Germany, (Regina Jutz)

* In addition to the items documented in the source questionnaire a set of standard background variables is collected with each ISSP module; see https://www.gesis.org/issp/home/issp-background-variables/.

Outline of thematic content in ISSP 2021 Health and Health Care Module

Topic	Question numbers	Old items	New items	Back-ground	Optional
Health Status	Q1, Q16a-Q16e, Q24, Q25, Q26a, Q26b	10			
Health Behaviour	Q22, Q23a-Q23c	4			
Beliefs about Health	Q8a-Q8d, Q9	5			
Health Insurance, Access and Utilization	Q17a, Q17b, Q18a-Q18c	5(6)		Q28	
Right to Health Care - Equality	Q3, Q5, Q6a-Q6b, Q7a-Q7d	4	4		
Trust and Confidence in the Health Care system	Q2, Q10a-Q10c	4			
Health Care System – Performance and satisfaction	Q4a-Q4c, Q19, Q20, Q21a, Q21b	7			
eHealth and ePatients (NT)	Q11, Q12a-Q12c Q13a, Q13b, Q14a-Q14c		9		
Attitudes towards vaccination (NT)	Q15a-Q15b		2		
COVID-19	Q27a-27e		5		
Optional items – COVID-19	Q29a-Q29c, Q30a, Q30b, Q31, Q32, Q33				8
Optional items – Stigma	Q34a, Q34b				2
**TOTAL**		**39(40)**	**20**	**1**	**10**

Note: NT = New topic for ISSP 2021; question numbers refer specifically to the numbering employed in this document. Q28 about health insurance is proposed as a compulsory background variable following a module-specific obligatory coding scheme.

ISSP 2021 Module on Health and Health Care

Final draft questionnaire April 2020

1 All notes which are not part of the questionnaire are enclosed in pointed brackets,
e.g. **<ISSP 2002>**.

2 Where items have previously been used in other ISSP modules this is noted. Countries should ensure that their translation for these questions matches that used on any previous occasion(s) when the questions were fielded.

3 Where [country] appears in the question text, members should insert the name of their country.

<ISSP 2002: Q17, ISSP 2011: Q1, ISSP 2012: Q24>

1. If you were to consider your life in general these days, how happy or unhappy would you say you are, on the whole?

*PLEASE TICK **ONE** BOX ONLY*

Completely happy	☐	(1)
Very happy	☐	(2)
Fairly happy	☐	(3)
Neither happy nor unhappy	☐	(4)
Fairly unhappy	☐	(5)
Very unhappy	☐	(6)
Completely unhappy	☐	(7)
Can’t choose	☐	(-8)

<ISSP 2011: Q2b>

2. In general, how much confidence do you have in the health care system in [country]?

*PLEASE TICK **ONE** BOX ONLY*

Complete confidence	☐	(1)
A great deal of confidence	☐	(2)
Some confidence	☐	(3)
Very little confidence	☐	(4)
No confidence at all	☐	(5)
Can't choose	☐	(-8)

<ISSP 2011: Q5>
3. Is it fair or unfair that people with higher incomes can afford better **health care** than people with lower incomes?

*PLEASE TICK **ONE** BOX ONLY*

Very fair	☐	(1)
Somewhat fair	☐	(2)
Neither fair nor unfair	☐	(3)
Somewhat unfair	☐	(4)
Very unfair	☐	(5)
Can't choose	☐	(-8)

<ISSP 2011: Q6b/c/d>
4. How much do you agree or disagree with the following statements?

PLEASE TICK ONE BOX ON EACH LINE

	Strongly agree	Agree	Neither agree nor disagree	Disagree	Strongly disagree	Can't choose
a. People use health care services more than necessary.	☐	☐	☐	☐	☐	☐
b. The government should provide only limited health care services.	☐	☐	☐	☐	☐	☐
c. In general, the health care system in [country] is inefficient.	☐	☐	☐	☐	☐	☐
	(1)	(2)	(3)	(4)	(5)	(-8)

<TN: in answer category b, "limited" can also be understood as basic>

<ISSP 2011: Q7>
5. How willing would you be to pay higher taxes to improve the level of health care for all people in [country]?

*PLEASE TICK **ONE** BOX ONLY*

Very willing	☐	(1)
Fairly willing	☐	(2)
Neither willing nor unwilling	☐	(3)
Fairly unwilling	☐	(4)
Very unwilling	☐	(5)
Can't choose	☐	(-8)

<ISSP 2011: Q9a/b >
6. How much do you agree or disagree with the following statements?

*PLEASE TICK **ONE** BOX ON EACH LINE*

People should have access to publicly funded health care even if they…	Strongly agree	Agree	Neither agree nor disagree	Disagree	Strongly disagree	Can't choose
a. do not hold citizenship of [country].	☐	☐	☐	☐	☐	☐
b. behave in ways that damage their health.	☐	☐	☐	☐	☐	☐
	(1)	(2)	(3)	(4)	(5)	(-8)

<NEW>

7. In [country], do you think it is easier or harder to get access to health care …

*PLEASE TICK **ONE** BOX ON EACH LINE*

	Much easier	Some-what easier	About the same	Some-what harder	Much harder	Can't choose
a. for rich people than for poor people	☐	☐	☐	☐	☐	☐
b. for old people than for young people	☐	☐	☐	☐	☐	☐
c. for women than for men	☐	☐	☐	☐	☐	☐
d. for citizens of [country] than for people who do not hold [country´s] citizenship	☐	☐	☐	☐	☐	☐
	(1)	(2)	(3)	(4)	(5)	(-8)

<ISSP 2011: Q11a-d >

8. Severe health problems may have many causes. How much do you agree or disagree with the following statements?

*PLEASE TICK **ONE** BOX ON EACH LINE*

People suffer from severe health problems…	Strongly agree	Agree	Neither agree nor disagree	Disagree	Strongly disagree	Can't choose
a. because they behaved in ways that damaged their health.	☐	☐	☐	☐	☐	☐
b. because of the environment they are exposed to at work or where they live.	☐	☐	☐	☐	☐	☐
c. because of their genes.	☐	☐	☐	☐	☐	☐
d. because they are poor.	☐	☐	☐	☐	☐	☐
	(1)	(2)	(3)	(4)	(5)	(-8)

<ISSP 2011: 15a>

9. To what extent do you agree or disagree with the following statement: [Alternative/traditional/folk] medicine provides better solutions for health problems than [mainstream/Western conventional] medicine?

*PLEASE TICK **ONE** BOX ONLY*

Strongly agree	☐	(1)
Agree	☐	(2)
Neither agree nor disagree	☐	(3)
Disagree	☐	(4)
Strongly disagree	☐	(5)
Can't choose	☐	(-8)

<TN: “Alternative/traditional/folk medicine”: Each country should choose the term that most appropriately refers to medical and health care practices and products, which are not currently part of mainstream Western medicine.
"mainstream/Western conventional medicine": Each country should choose the term that most appropriately refers to allopathic mainstream Western medicine.
This translation note is also relevant to questions 17 and 21>

<ISSP 2011: Q16a/c/d>

10. How much do you agree or disagree with the following statements about doctors in general in [country]?

*PLEASE TICK **ONE** BOX ON EACH LINE*

	Strongly agree	Agree	Neither agree nor disagree	Disagree	Strongly disagree	Can't choose
a. All things considered, doctors can be trusted.	☐	☐	☐	☐	☐	☐
b. The medical skills of doctors are not as good as they should be.	☐	☐	☐	☐	☐	☐
c. Doctors care more about their earnings than about their patients.	☐	☐	☐	☐	☐	☐
	(1)	(2)	(3)	(4)	(5)	(-8)

<NEW>

11. During the past 12 months, how often, if at all, did you use the internet on any device (such as computers, tablets and smartphones) to look for health or medical information for yourself or someone else?

PLEASE TICK **ONE** BOX ONLY

Several times a day	☐	(1)
Once a day	☐	(2)
Several times a week	☐	(3)
Several times a month	☐	(4)
Several times a year	☐	(5)
Never or almost never	☐	(6)
Can't choose	☐	(-8)
I do not have access to the internet	☐	(-4) Skip to Q14

<NEW>
12. During the past 12 months, how often, if at all, have you used the internet to look for information on the following topics?

*PLEASE TICK **ONE** BOX ON EACH LINE*

	Never	Seldom	Sometimes	Often	Very often	Can't choose
a. Information on healthy lifestyle?	☐	☐	☐	☐	☐	☐
b. Information related to anxiety, stress, or similar problems?	☐	☐	☐	☐	☐	☐
c. Information on vaccinations?	☐	☐	☐	☐	☐	☐
	(1)	(2)	(3)	(4)	(5)	(-8)

<TN: Healthy lifestyle: behaviours that help to keep or improve people´s physical and mental health.

<NEW>
13. To what extent do you agree or disagree with the following statements?
During the past 12 months, information on the internet…

*PLEASE TICK **ONE** BOX ON EACH LINE*

	Strongly agree	Agree	Neither agree nor disagree	Disagree	Strongly disagree	Can't choose
a. affected my health behaviour in a positive way.	☐	☐	☐	☐	☐	☐
b. helped me understand what a doctor tried to explain to me.	☐	☐	☐	☐	☐	☐
	(1)	(2)	(3)	(4)	(5)	(-8)

<NEW>
14. To what extent do you agree or disagree with the following statements?

*PLEASE TICK **ONE** BOX ON EACH LINE*

	Strongly agree	Agree	Neither agree nor disagree	Disagree	Strongly disagree	Can't choose
a. The internet is useful to help people decide if their symptoms are serious enough to go to the doctor.	☐	☐	☐	☐	☐	☐
b. The internet is useful to check that the doctor is giving people appropriate advice.	☐	☐	☐	☐	☐	☐
c. It is **not** easy to distinguish between reliable and unreliable health information on the internet.	☐	☐	☐	☐	☐	☐
	(1)	(2)	(3)	(4)	(5)	(-8)

<NEW>

15. How much do you agree or disagree with the following statements about vaccinations?

*PLEASE TICK **ONE** BOX ON EACH LINE*

	Strongly agree	Agree	Neither agree nor disagree	Disagree	Strongly disagree	Can't choose
a. Overall, vaccinations do more harm than good.	☐	☐	☐	☐	☐	☐
b. It is better to develop immunity by getting ill than having a vaccination.	☐	☐	☐	☐	☐	☐
	(1)	(2)	(3)	(4)	(5)	(-8)

<ISSP 2011: Q17a-e>

16. During the **past 4 weeks** how often**…**

*PLEASE TICK **ONE** BOX ON EACH LINE*

	Never	Seldom	Sometimes	Often	Very often	Can't choose
a. have you had difficulties with work or household activities because of health problems?	☐	☐	☐	☐	☐	☐
b. have you had bodily aches or pains?	☐	☐	☐	☐	☐	☐
c. have you felt unhappy and depressed?	☐	☐	☐	☐	☐	☐
d. have you lost confidence in yourself?	☐	☐	☐	☐	☐	☐
e. have you felt you could not overcome your problems?	☐	☐	☐	☐	☐	☐
	(1)	(2)	(3)	(4)	(5)	(-8)

<ISSP 2011: Q18a/b>

17. During the past 12 months, how often did you visit or were visited by...

*PLEASE TICK **ONE** BOX ON EACH LINE*

	Never	Seldom	Sometimes	Often	Very often	Can't choose
a. a doctor?	☐	☐	☐	☐	☐	☐
b. an [alternative/traditional /folk] health care practitioner?	☐	☐	☐	☐	☐	☐
	(1)	(2)	(3)	(4)	(5)	(-8)

<TN: “Alternative/traditional/folk medicine”: Each country should choose the term that most appropriately refers to medical and health care practices and products, which are not currently part of mainstream Western medicine.
This translation note is also relevant to question 21>

<ISSP 2011: Q20a/b/d>

18. During the past 12 months did it ever happen that you did **not** get the medical treatment you needed because…

*PLEASE TICK **ONE** BOX ON EACH LINE*

	Yes	No	Did not need medical treatment
a. you could not pay for it?	☐	☐	☐
b. you could not take the time off work or had other commitments?	☐	☐	☐
c. the waiting list was too long?	☐	☐	☐
	(1)	(2)	(-4)

<ISSP 2011: Q21a>

19. How likely is it that if you become seriously ill, you would get or not get the best treatment available in [country]

*PLEASE TICK **ONE** BOX ONLY*

It's certain I would get	☐	(1)
It's likely I would get	☐	(2)
Equal chance of getting or not getting	☐	(3)
It's likely I would not get	☐	(4)
It's certain I would not get	☐	(5)
Can't choose	☐	(-8)

<ISSP 2011: Q22>

20. In general, how satisfied or dissatisfied are you with the health care system in [country]?

*PLEASE TICK **ONE** BOX ONLY*

Completely satisfied	☐	(1)
Very satisfied	☐	(2)
Fairly satisfied	☐	(3)
Neither satisfied nor dissatisfied	☐	(4)
Fairly dissatisfied	☐	(5)
Very dissatisfied	☐	(6)
Completely dissatisfied	☐	(7)
Can’t choose	☐	(-8)

<ISSP 2011: Q23a/b >

21. How satisfied or dissatisfied were you with the treatment you received…

*PLEASE TICK **ONE** BOX ON EACH LINE*

	Completely satisfied	Very satisfied	Fairly satisfied	Neither satisfied nor dissatisfied	Fairly dissatisfied	Very dissatisfied	Completely dissatisfied	Does not apply	Can't choose
a. when you last visited a doctor?	☐	☐	☐	☐	☐	☐	☐	☐	☐
b. when you last visited an [alternative /traditional/ folk] health care practitioner?	☐	☐	☐	☐	☐	☐	☐	☐	☐
	(1)	(2)	(3)	(4)	(5)	(6)	(7)	(-4)	(-8)

<TN: By "alternative/traditional/folk" health care practitioners we mean someone who was not trained in mainstream Western medicine or does not practice it. Please use the term most appropriate for your country.>

<ISSP 2011: Q24>

22. Do you smoke cigarettes, and if so about how many cigarettes a day?

*PLEASE TICK **ONE** BOX ONLY*

Do not smoke and never did	☐	(1)
Do not smoke now but smoked in the past	☐	(2)
Smoke 1-5 cigarettes per day	☐	(3)
Smoke 6-10 cigarettes per day	☐	(4)
Smoke 11-20 cigarettes per day	☐	(5)
Smoke 21-40 cigarettes per day	☐	(6)
Smoke more than 40 cigarettes per day	☐	(7)
Can't choose	☐	(-8)

<ISSP 2011: Q25a-c>

23. How often do you…

*PLEASE TICK **ONE** BOX ON EACH LINE*

	Never	Once a month or less often	Several times a month	Several times a week	Daily	Can't choose
a. drink 4 or more alcoholic drinks on the same day?	☐	☐	☐	☐	☐	☐
b. do physical activity for at least 20 minutes that makes you sweat or breathe more heavily than usual?	☐	☐	☐	☐	☐	☐
c. eat fresh fruit or vegetables?	☐	☐	☐	☐	☐	☐
	(1)	(2)	(3)	(4)	(5)	(-8)

<ISSP 2007: Q17, ISSP 2011: Q26, ISSP 2017: Q27>

24. In general, would you say your health is …

*PLEASE TICK **ONE** BOX ONLY*

excellent ☐ (1)

very good ☐ (2)

good ☐ (3)

fair ☐ (4)

poor ☐ (5)

Can't choose ☐ (-8)

<TN: This refers to both physical and mental health.>

<ISSP 2011: Q27>

25. Do you have a long-standing illness, a chronic condition, or a disability?

*PLEASE TICK **ONE** BOX ONLY*

Yes ☐ (1)

No ☐ (2)

<ISSP 2011: Q28>

26. What is your…

PLEASE ENTER

a. height: ________cm I don't know ☐ (-8)

b. weight: ________kg I don't know ☐ (-8)

<Countries not using metric height and weight:
please use local units and convert to metric units in the data file you deposit to the Archive>

<NEW – wording based on ISSP 2016: Q11, Q13, Q14>

27. Do you think the [country] government should or should not have the right to do the following at times of severe epidemics?

*PLEASE TICK **ONE** BOX ON EACH LINE*

	Definitely should have the right	Probably should have the right	Probably should not have the right	Definitely should not have the right	Can't choose
a. Shut down businesses and places of employment.	☐	☐	☐	☐	☐
b. Demand that people stay at home.	☐	☐	☐	☐	☐
c. Use digital (mobile phone) surveillance to track infected people.	☐	☐	☐	☐	☐
d. Require people to wear face masks.	☐	☐	☐	☐	☐
e. Ban public gatherings.	☐	☐	☐	☐	☐
	(1)	(2)	(3)	(4)	(-8)

<TN ”in government” as in who is in executive office/power.>

<TN: ‘Demand’ in the sense of ‘impose’. This can be done through the passing of a law, but also through a government ordinance.>

REQUIRED ITEM WITH BACKGROUND QUESTIONS

<Note to ISSP members: Q28 is a compulsory background variable following a module-specific obligatory coding scheme. The question should be asked in a way (wording and categories) relevant to your country. However, please include the category "Have no health insurance" even if only a small number of your respondents are likely to place themselves in this category. The answers of each respondent should be coded into one of the following categories.>

<ISSP 2011: Q29>

28. What kind of health insurance do you have?

Have no health insurance	(1)	☐
National/public health insurance (including coverage by public welfare) (A)	(2)	☐
Private insurance (B)	(3)	☐
Employer/union based insurance (C)	(4)	☐
National/public health insurance and private/complementary insurance (A+B)	(5)	☐
Public/national and employer/union based insurance (A+C)	(6)	☐
Employer/union based and private/complementary insurance (B+C)	(7)	☐
Employer/union based, private/complementary and national/public health insurance (A+B+C)	(8)	☐
Other (specify)_______________	(9)	☐

<National/public health insurance - a system that provides a publicly defined level of health services. <u>The label should be adapted to national terms.</u>
TN: Coverage by public welfare system - health insurance provided by the state to those who are on welfare and cannot afford to pay. This refers to category 2. >

OPTIONAL ITEMS

<NEW– wording based on ISSP 2016: Q11, Q13, Q14>

29. Do you think the [country] government should or should not have the right to do the following at times of severe epidemics?

*PLEASE TICK **ONE** BOX ON EACH LINE*

	Definitely should have the right	Probably should have the right	Probably should not have the right	Definitely should not have the right	Can't choose
a. Place people known to carry the disease in isolation.	☐	☐	☐	☐	☐
b. Suspend compulsory education and close schools and kindergartens.	☐	☐	☐	☐	☐
c. Close borders to other countries.	☐	☐	☐	☐	☐
	(1)	(2)	(3)	(4)	(-8)

<TN ”in government” as in who is in executive office/power.>

<NEW>

30. Did the way the Covid-19 pandemic was handled in [country] increase or decrease your confidence in…

*PLEASE TICK **ONE** BOX ON EACH LINE*

	Increased it a lot	Increased it a little	Neither increased it nor decreased it	Decreased it a little	Decreased it a lot	Can't choose
a. the health care system?	☐	☐	☐	☐	☐	☐
b. the government?	☐	☐	☐	☐	☐	☐
	(1)	(2)	(3)	(4)	(5)	(-8)

<NEW>
31. Considering your work activity before the Covid-19 pandemic and at present, which of the following statements best describes your employment situation…

*PLEASE TICK **ONE** BOX ONLY*

I did not have a job before the pandemic and I do not have a job now	☐	(1)
I have the same job as before the pandemic	☐	(2)
I lost my job due to the pandemic and now have a new job	☐	(3)
I lost my job due to the pandemic and could not find a new job	☐	(4)
I did not have a job before the pandemic and I do have a job now	☐	(5)
I changed or left my job for reasons unrelated to the pandemic	☐	(6)
Can't choose	☐	(-8)

<NEW – wording based on optional items in ISSP 2015: : O4/O5>
32. Thinking about the income of your household before the Covid-19 pandemic compared with now, would you say it has increased, decreased or stayed about the same?

*PLEASE TICK **ONE** BOX ONLY*

Increased a lot	☐	(1)
Increased a little	☐	(2)
Stayed about the same	☐	(3)
Decreased a little	☐	(4)
Decreased a lot	☐	(5)
Can't choose	☐	(-8)

<NEW >
33. Thinking about how often you met with your extended family and friends in person before the Covid-19 pandemic, would you say you now meet them in person more often, less often, or about the same?

*PLEASE TICK **ONE** BOX ONLY*

Much less often	☐	(1)
A little less often	☐	(2)
About the same	☐	(3)
A little more often	☐	(4)
Much more often	☐	(5)
Can't choose	☐	(-8)

<NEW>
34. There are different opinions about people that have certain health conditions. How much do you agree or disagree with the following statements?

*PLEASE TICK **ONE** BOX ON EACH LINE*

	Strongly agree	Agree	Neither agree nor disagree	Disagree	Strongly disagree	Can't choose
a. Most people become very overweight because they are lazy.	☐	☐	☐	☐	☐	☐
b. Most people who tested positive for Covid-19 contracted the virus because they were careless.	☐	☐	☐	☐	☐	☐
	(1)	(2)	(3)	(4)	(5)	(-8)

Hungary
ISSP 2021 – Health and Health Care II
Questionnaire

sorszám

ÖSSZESEN HÁNY FŐS A HÁZTARTÁS? ☐☐ fős

A KÉRDEZETT SZÜLETÉSI ÉVE: ☐☐☐☐

OMNIBUSZ

2021/04

BELSŐ MUNKAANYAG

A válaszadás önkéntes!

Település neve: .... Budapesten kerület: ☐☐

Kijelentem, hogy a kérdezés szabályainak megfelelően jártam el. Az általam kezelt és felvett adatokat bizalmasan kezelem, azokat csak a kutatásban illetékes személynek adom át,

Kérdező aláírása... ☐☐☐☐☐

kérdezői igazolványszám

KÉRDEZÉS KEZDETE: 2021. hónap ……….. nap…….. óra…… perctől

Adatkezelési hozzájárulás

A TÁRKI Társadalomkutatási Intézet Zrt negyedévenként folytatott Omnibusz kutatásaiban megrendelőink megbízásából egy sor változatos témával kapcsolatban kérdezzük válaszadóink véleményét.
A válaszadás önkéntes és az információkat bizalmasan kezeljük. Az interjú során a válaszait a számítógépen rögzítem. Az Ön címét és nevét szigorúan elkülönítve tároljuk az interjú alatt megadott információktól. Ha nem ad engedélyt arra, hogy a későbbiekben is felkereshessük, az Ön elérhetőségét és nevét csak a jelen kutatást követő fél évig, kizárólag a kérdezőink munkájának ellenőrzése céljából tároljuk. Az adatokat nevek és címek nélkül összesítve elemezzük kutatási célból, anélkül, hogy az egyes kutatók ismernék az Ön személyét. A kutatás eredményeit csak anonim formában mutatjuk be.

Ha valamelyik kérdésre nem szeretne válaszolni, ezt bármikor jelezheti. A részvétel elutasítása semmilyen hátrányt nem jelent Önnek. A beleegyezését bármikor visszavonhatja a jövőben.

Beleegyezik a kutatásban való részvételbe?

1 - Igen, a válaszadó beleegyezett a részvételbe.
2 - Nem, a válaszadó elutasította a részvételt. Az interjú nem lehetséges. --> Interjú vége

KÉRDEZŐ: ha a válaszadó beleegyezik az interjúba, írasd vele alá a címkártya „Adatkezelési hozzájárulás” nyilatkozatát, mert enélkül a kérdőív nem fogadható el!

1. Mi az Ön legmagasabb iskolai végzettsége?

1 – kevesebb, mint 8 általános
2 – 8 általános
3 – szakmunkásképző; szakképzés érettségi nélkül
4 – szakközépiskolai érettségi; szakképzést követő érettségi
5 – gimnáziumi érettségi
6 – érettségit követő, felsőfokra nem akkreditált szakképzés; középfokú technikum
7 – akkreditált felsőfokú szakképzés; felsőfokú technikum
8 – főiskola / BA / BSc
9 – egyetem / MA / MSc
X –

2. Összes iskolai tanulmányait figyelembe véve, hány évet tanult Ön bármilyen nappali tagozatos iskolában? Kérem az évismétléseket ne számolja bele!

……………….évet
99 – NT X –

3. Összesen Önnel együtt hányan élnek Önök egy háztartásban?

.... fő X –

4. Most a háztartás tagjaira vonatkozóan tennék fel néhány kérdést. Kezdjük Önnel a sort!
Kit tekintenek Önök háztartásfőnek?

1 – a kérdezett választott háztartásfőt
2 – a kérdezett nem választott háztartásfőt ⟶ HA A KÉRDEZETT NEM TUD DÖNTENI VÁLASSZ TE HÁZTARTÁSFŐT!
0 – egyedül él a kérdezett
X –

A HÁZTARTÁSFŐ

A legidősebb 18-62 év közötti férfi
hiányában:
A legidősebb 18-62 év közötti nő
hiányában:
A legidősebb 62 év feletti férfi
hiányában:
A legidősebb 62 év feletti nő

	neme		születésének … éve NÉGY SZÁMJEGYGYEL	születésének … hónapja KÉT SZÁMJEGYGYEL	A háztartásfőhöz viszonyított családi állása
	férfi	nő			KÓD
**KÉRDEZETT**	1	2	X –	X –	X –
**2.**	1	2	X –	X –	X –
**3.**	1	2	X –	X –	X –
**4.**	1	2	X –	X –	X –
**5.**	1	2	X –	X –	X –
**6.**	1	2	X –	X –	X –
**7.**	1	2	X –	X –	X –

HÁZTARTÁSFŐHÖZ VISZONYÍTOTT CSALÁDI ÁLLÁSA:

1 – háztartásfő
2 – házastársa
3 – élettársa
4 – gyermeke
5 – szülője, anyósa, apósa
6 – egyéb rokon
8 – nem rokon

**8.**	1	2	X –	X –		X –
**9.**	1	2	X –	X –		X –

5. Mi az Ön családi állapota?

1 – nőtlen, hajadon
2 – házas és együtt élnek → 7 - RE
3 – házas és külön élnek
4 – elvált
5 – özvegy
X –

6. Van Önnek jelenleg élettársa vagy partnere?

1 – igen, élettársa, partnere van, együtt élnek
2 – partnere, élettársa van, de nem élnek együtt
3 – nincs se élettársa, se partnere → 7 - RE
X –

6.b. Bejegyeztették-e az élettársi kapcsolatukat közjegyző előtt?

1 – igen
2 – nem
X –

MINDENKITŐL!

7. Ez a település, ahol Ön lakik …

1 – nagyváros
2 – nagyváros környéke,
3 – kisváros
4 – falu, község vagy
5 – tanya?

9 – NT X –

8. Melyik országban született az Ön édesapja?

ÍRD LE, UTÁNA KÓDOLD!

Ha az az ország már nem létezik, akkor a jelenlegi országot írd be!

....

KÓD:

01 – Magyarország	09 - Ausztria
02 – Ukrajna	10 - Németország
03 – Románia	11 - Oroszország
04 – Szlovákia	12 - Lengyelország
05 – Csehország	13 - Egyéb európai ország
06 – Szerbia	14 - Egyéb nem európai ország
07 – Horvátország	88 - Megtagadja a választ
08 – Szlovénia	99 - NT

9. Melyik országban született az Ön édesanyja?

ÍRD LE, UTÁNA KÓDOLD!

....

KÓD:

Ha az az ország már nem létezik, akkor a jelenlegi országot írd be!

01 – Magyarország	09 - Ausztria
02 – Ukrajna	10 - Németország
03 – Románia	11 - Oroszország
04 – Szlovákia	12 - Lengyelország
05 – Csehország	13 - Egyéb európai ország
06 – Szerbia	14 - Egyéb nem európai ország
07 – Horvátország	88 - Megtagadja a választ
08 – Szlovénia	99 - NT

10. Ön jelenleg dolgozik / nyugdíjas?

01 – alkalmazott	06 – szülési szabadságon, gyeden, gyesen, gyeten var
02 – önálló, vállalkozó, saját vállalkozásában alkalmazott	07 – háztartásbeli, családját látja el, főállású anya
03 – alkalmi munkát, megbízásokat vállal	08 – tanuló
04 – munkanélküli	09 – egyéb
05 – nyugdíjas (öregségi, rokkant, özvegyi)	99 – NT X –

11. HA ALKALMAZOTT! (10. Kérdés 1-es kód)

Ön a saját családjának vállalkozásának alkalmazottja-e?

1 – igen 2 – nem X –

12. HA ALKALMAZOTT! (10. Kérdés 1-es kód)

Ön jelenleg közmunkás?

1 – igen 2 – nem X –

13. HA NYUGDÍJAS! (10. Kérdés 5-ös kód)

Ön …

1 – öregségi, özvegyi nyugdíjas, vagy
2 – rokkant nyugdíjas, rehabilitációs járadékot kap?
X –

14. HA JELENLEG TANULÓ! (10. kérdés: 8-as kód)

Gyakornokként, ipari tanulóként dolgozik-e Ön jelenleg?

1 – igen
2 – nem
X —

MINDENKITŐL!

15. Keres-e Ön jelenleg munkát?

1 – igen
2 – nem
X —

16. Mi (volt) a (legutolsó) foglalkozása, beosztása?

....
....

RÉSZLETESEN ÍRD LE, ÉS A VÁLASZ ALAPJÁN A HELYSZÍNEN TISZTÁZD A BESOROLÁST!

ÖNÁLLÓK, VÁLLALKOZÓK (tulajdonos, vagy résztulajdonos)
- 01 – gazdálkodó, őstermelő (mezőgazdaságban)
- 02 – fizikai munkát is végző kisvállalkozó (iparos, kereskedő)
- 03 – szellemi szabadfoglalkozású
- 04 – egyéb

ALKALMAZOTTAK (nem a saját vállalkozásában dolgozik)

VEZETŐ:
- 06 – felső vezető (osztályvezető felett)
- 07 – középszintű vezető (osztályvezető)
- 08 – alsó vezető (osztályvezető alatt)
- 09 – közvetlen termelésirányító („kék galléros”, fizikai beosztottakkal, pl. művezető)

folytatás:

ALKALMAZOTTAK (nem a saját vállalkozásában dolgozik)

SZELLEMI:
- 10 – diplomához kötött
- 11 – egyéb szellemi (diploma nélkül)

FIZIKAI
- 12 – szakmunkás (nem mezőgazdasági)
- 13 – betanított munkás (nem mezőgazdasági)
- 14 – segédmunkás (nem mezőgazdasági)
- 15 – **mezőgazdasági** fizikai

00 – sosem dolgozott → 24 - RE

99 – NT
X –

17. ÖNÁLLÓTÓL, VEZETŐTŐL! (előző kérdés 01 - 09-es kód)

Hány alkalmazottja / beosztottja van (volt)?

.......….. alkalmazott / beosztott
000 – nincs, nem volt alkalmazottja
999 – NT X –

18. HA DOLGOZIK! (10. kérdés 1-2-3-as kód)

Körülbelül hány órát tölt Ön átlagosan munkával egy héten? Kérem, számítsa bele a túlórákat és az esetleges mellékállásait is!

…………..órát
999 – NT X –

19-23. HA DOLGOZIK /DOLGOZOTT!

19. Miből áll (állt) az Ön munkája?

ÍRD LE!

9 – NT X –

20. A munkahelyén általában mit termelnek, mit csinálnak, illetve milyen szolgáltatásokat nyújtanak?

ÍRD LE!

9 – NT X –

21. Ön …
1 – köztisztviselő, közalkalmazott,
2 – állami, önkormányzati vállalat alkalmazottja, vagy
3 – más helyen dolgozik / dolgozott?
9 – NT X –

22. Mi (volt) az ön munkáltatójának gazdálkodási formája?...

01 – egyéni vállalkozás
02 – Bt. - Betéti társaság
03 – Kft – Korlátolt felelősségű társaság
04 – Kkt – Közkereseti társaság
05 – Zrt – Zártkörű részvénytársaság
06 – Nyrt - Nyilvánosan működő részvénytársaság
07 – Kht – Közhasznú társaság
08 – Szövetkezet
09 – Alapítvány, Közhasznú Alapítvány
10 – Államigazgatás, önkormányzat (ideértve a közoktatást, az állami felsőfokú oktatást és az állami egészségügyi intézményeket)
77 – egyéb, éspedig: ………….
99 – NT X –

23. Mi (volt) a munkáltatójának főtevékenysége?

01 – mezőgazdaság, erdőgazdálkodás, halászat
02 – bányászat, kőfejtés
03 – feldolgozóipar
04 – villamosenergia-, gáz-, gőzellátás, légkondicionálás
05 – vízellátás, szennyvíz gyűjtése, kezelése, hulladékgazdálkodás, szennyeződésmentesítés
06 – építőipar
07 – kereskedelem, gépjárműjavítás
08 – szállítás, raktározás
09 – szálláshely-szolgáltatás, vendéglátás
10 – információ, kommunikáció
11 – pénzügyi, biztosítási tevékenység
12 – ingatlanügyletek
13 – szakmai, tudományos, műszaki tevékenység
14 – adminisztratív és szolgáltatást támogató tevékenység
15 – közigazgatás, védelem; kötelező társadalombiztosítás
16 – oktatás
17 – humán-egészségügyi, szociális ellátás
18 – művészet, szórakoztatás, szabadidő
19 – egyéb szolgáltatás
20 – háztartás munkaadói tevékenysége; termék előállítása, szolgáltatás végzése saját fogyasztásra
21 – területen kívüli szervezet (pl. nemzetközi szervezet, nagykövetség… stb.)

99 – NT X –

24-36. HA HÁZAS VAGY ÉLETTÁRSSA, PARTNERE VAN! (5. Kérdés, 2-3-as kód VAGY 6. Kérdés, 1-2-es kód)

24. Az Ön házastársa / élettársa jelenleg dolgozik / nyugdíjas?

01 – alkalmazott
02 – önálló, vállalkozó, saját vállalkozásában alkalmazott
03 – alkalmi munkát, megbízásokat vállal
04 – munkanélküli
05 – nyugdíjas (öregségi, rokkant, özvegyi)
06 – szülési szabadságon, gyeden, gyesen, gyeten van
07 – háztartásbeli, családját látja el, főállású anya
08 – tanuló
09 – egyéb
99 – NT X –

25. Mi az Ön házastársának/partnerének a legmagasabb iskolai végzettsége?

1 – kevesebb, mint 8 általános
2 – 8 általános
3 – szakmunkásképző; szakképzés érettségi nélkül
4 – szakközépiskolai érettségi; szakképzést követő érettségi
5 – gimnáziumi érettségi
6 – érettségit követő, felsőfokra nem akkreditált szakképzés; középfokú technikum
7 – akkreditált felsőfokú szakképzés; felsőfokú technikum
8 – főiskola / BA / BSc
9 – egyetem / MA / MSc
X –

26. HA ALKALMAZOTT A HÁZAS/ ÉLETTÁRSA! (24. Kérdés 1-es kód)

Az Ön házastársa / élettársa a saját családjának vállalkozásának alkalmazottja-e?

1 – igen 2 – nem X –

27. HA ALKALMAZOTT A HÁZAS/ ÉLETTÁRSA! (24. Kérdés 1-es kód)

Az Ön élettársa jelenleg közmunkásként dolgozik?

1 – igen 2 – nem X –

28. HA NYUGDÍJAS A HÁZAS/ ÉLETTÁRSA! (24. Kérdés 5-ös kód)

Az Ön házastársa / élettársa …

1 – öregségi, özvegyi nyugdíjas, vagy
2 – rokkant nyugdíjas, rehabilitációs járadékot kap?
X –

29. HA JELENLEG TANULÓ A HÁZAS/ ÉLETTÁRSA! (24. kérdés: 8-as kód)

Gyakornokként, ipari tanulóként dolgozik-e jelenleg?

1 – igen
2 – nem
X —

MINDENKITŐL!

30. Keres-e az Ön házastársa / élettársa jelenleg munkát?

1 – igen
2 – nem
X —

31. Mi (volt) a (legutolsó) foglalkozása, beosztása az Ön házas/ élettársának?

....

RÉSZLETESEN ÍRD LE, ÉS A VÁLASZ ALAPJÁN A HELYSZÍNEN TISZTÁZD A BESOROLÁST!

ÖNÁLLÓK, VÁLLALKOZÓK (tulajdonos, vagy résztulajdonos)

01 – gazdálkodó, őstermelő (mezőgazdaságban)
02 – fizikai munkát is végző kisvállalkozó (iparos, kereskedő)
03 – szellemi szabadfoglalkozású
04 – egyéb

ALKALMAZOTTAK (nem a saját vállalkozásában dolgozik)

VEZETŐ: 06 – felső vezető (osztályvezető felett)
07 – középszintű vezető (osztályvezető)
08 – alsó vezető (osztályvezető alatt)
09 – közvetlen termelésirányító („kék galléros", fizikai beosztottakkal, pl. művezető)

folytatás:

ALKALMAZOTTAK (nem a saját vállalkozásában dolgozik)

SZELLEMI: 10 – diplomához kötött
11 – egyéb szellemi (diploma nélkül)

FIZIKAI 12 – szakmunkás (nem mezőgazdasági)
13 – betanított munkás (nem mezőgazdasági)
14 – segédmunkás (nem mezőgazdasági)
15 – **mezőgazdasági** fizikai

00 – sosem dolgozott → 36 - RA
99 – NT
X –

32. HA A HÁZAS/ ÉLETTÁRSA ÖNÁLLÓ, VEZETŐ! (előző kérdés 01 - 09-es kód)

Hány alkalmazottja / beosztottja van (volt)?

.... alkalmazott / beosztott
000 – nincs, nem volt alkalmazottja
999 – NT X –

33. HA DOLGOZIK A HÁZAS/ ÉLETTÁRSA! (24. kérdés 1-2-3-as kód)

Körülbelül hány órát tölt az Ön házastársa / élettársa átlagosan munkával egy héten? Kérem, számítsa bele a túlórákat és az esetleges mellékállásait is!

……………órát
999 – NT X –

34 – 36. HA DOLGOZIK /DOLGOZOTT A HÁZAS/ ÉLETTÁRSA!

34. Miből áll (állt) az az Ön házas / élettársának a munkája?

ÍRD LE!

9 – NT X –

35. Az Ön házas / élettársa munkahelyén mit termelnek, mit csinálnak, illetve milyen szolgáltatásokat nyújtanak?

ÍRD LE!

---

9 – NT X –

36. Mi (volt) a munkáltatójának főtevékenysége?

01 – mezőgazdaság, erdőgazdálkodás, halászat
02 – bányászat, kőfejtés
03 – feldolgozóipar
04 – villamosenergia-, gáz-, gőzellátás, légkondicionálás
05 – vízellátás, szennyvíz gyűjtése, kezelése, hulladékgazdálkodás, szennyeződésmentesítés
06 – építőipar
07 – kereskedelem, gépjárműjavítás
08 – szállítás, raktározás
09 – szálláshely-szolgáltatás, vendéglátás
10 – információ, kommunikáció
11 – pénzügyi, biztosítási tevékenység
12 – ingatlanügyletek
13 – szakmai, tudományos, műszaki tevékenység
14 – adminisztratív és szolgáltatást támogató tevékenység
15 – közigazgatás, védelem; kötelező társadalombiztosítás
16 – oktatás
17 – humán-egészségügyi, szociális ellátás
18 – művészet, szórakoztatás, szabadidő
19 – egyéb szolgáltatás
20 – háztartás munkaadói tevékenysége; termék előállítása, szolgáltatás végzése saját fogyasztásra
21 – területen kívüli szervezet (pl. nemzetközi szervezet, nagykövetség… stb.)

99 – NT X –

MINDENKITŐL!

1. Megítélése szerint az Ön jelenlegi anyagi helyzete: nagyon jó; jó; nem is jó, nem is rossz; rossz vagy nagyon rossz?

5 – nagyon jó
4 – jó
3 – nem is jó, nem is rossz
2 – rossz
1 – nagyon rossz
9 – NT X -

2. Mit gondol, milyen lesz az Ön anyagi helyzete egy év múlva: sokkal jobb, mint most; valamivel jobb, mint most; ugyanilyen; valamivel rosszabb, mint most vagy sokkal rosszabb, mint most?

5 – sokkal jobb, mint most
4 – valamivel jobb, mint most
3 – ugyanilyen
2 – valamivel rosszabb, mint most
1 – sokkal rosszabb, mint most
9 – NT X -

3. Kérem, mondja meg, hogyan értékeli Ön az ország jelenlegi gazdasági helyzetét: nagyon jó; jó; nem is jó, nem is rossz; rossz vagy nagyon rossz?

5 – nagyon jó
4 – jó
3 – nem is jó, nem is rossz
2 – rossz
1 – nagyon rossz
9 – NT X -

4. Mire számít, a következő 12 hónapban az ország gazdasági helyzete sokkal jobb lesz, valamivel jobb lesz, nem változik, valamivel rosszabb lesz, vagy sokkal rosszabb lesz?

5 – sokkal jobb lesz
4 – valamivel jobb lesz
3 – nem változik
2 – valamivel rosszabb lesz
1 – sokkal rosszabb lesz
9 – NT X -

5. Kérem egy -5 és +5 terjedelmű skálán mondja meg, hogy Ön szerint ma Magyarországon általában véve jó vagy rossz irányba mennek a dolgok? A legmagasabb érték a +5, ami azt jelenti, hogy egyértelműen jó, a legalacsonyabb a –5, ami pedig azt jelenti, hogy teljes mértékben rossz irányba mennek a dolgok. A közbülső értékekkel árnyalhatja véleményét.

Teljes mértékben rossz										Egyértelműen jó
-5	-4	-3	-2	-1	0	+1	+2	+3	+4	+5

9 – NT X -

1. Mindent összevetve mit mondana, mennyire boldog ember Ön?

1 – Tökéletesen boldog,
2 – nagyon boldog,
3 – eléggé boldog,
4 – se nem boldog, se nem boldogtalan,
5 – eléggé boldogtalan,
6 – nagyon boldogtalan, vagy
7 – teljesen boldogtalan?

9 – NT X –

2. Általában véve mennyire bízik a magyar egészségügyi rendszerben?
[csak egy válasz]

1 – Teljesen megbízik
2 – Nagyban megbízik
3 – Valamennyire megbízik
4 – Nem nagyon bízik meg
5 – Egyáltalán nem bízik meg

9 – NT X –

3. Méltányos vagy sem, hogy a magasabb jövedelemmel rendelkező emberek jobb **egészségügyi ellátást** engedhetnek meg maguknak, mint az alacsonyabb jövedelmű emberek?
[csak egy válasz]

1 – Teljes mértékben méltányos
2 – Inkább méltányos
3 – Sem nem méltányos, sem nem méltánytalan
4 – Inkább méltánytalan
5 – Nagyon méltánytalan

9 – NT X –

4. Mennyire ért egyet a következő állításokkal? Teljesen egyetért, egyetért, egyet is ért meg nem is, nem ért egyet, vagy egyáltalán nem ért egyet?
[csak egy válasz soronként – KÉRDEZŐ: szükség esetén újra olvasd fel a válaszlehetőségeket!]

	Teljesen egyetért	Egyetért	Egyet is ért meg nem is	Nem ért egyet	Egyáltalán nem ért egyet	NT/NV
a. Az emberek a szükségesnél többet használják az egészségügyi szolgáltatásokat	1	2	3	4	5	9
b. Az államnak csak alapvető egészségügyi szolgáltatásokat kellene nyújtania	1	2	3	4	5	9
c. Általában véve a magyar egészségügyi rendszer működése nem hatékony	1	2	3	4	5	9

5. Mennyire volna Ön hajlandó magasabb adót fizetni annak érdekében, hogy mindenki számára javuljon az egészségügyi ellátás színvonala Magyarországon?
[csak egy válasz]

1 – Teljes mértékben igen
2 – Inkább igen
3 – Igen is meg nem is
4 – Inkább nem
5 – Egyáltalán nem

9 – NT X –

6. Mennyire ért egyet a következő állításokkal? Az embereknek hozzáférést kellene biztosítani az állami egészségügyi rendszerhez akkor is, ha…
[csak egy válasz soronként – KÉRDEZŐ: szükség esetén újra olvasd fel a válaszlehetőségeket!]

	Teljesen egyetért	Egyetért	Egyet is ért meg nem is	Nem ért egyet	Egyáltalán nem ért egyet	NT/NV
a. nem magyar állampolgárok	1	2	3	4	5	9
b. az egészségükre káros életmódot folytatnak	1	2	3	4	5	9

7. Mit gondol, Magyarországon könnyebb vagy nehezebb az egészségügyi ellátáshoz való hozzáférés…
[csak egy válasz soronként – KÉRDEZŐ: szükség esetén újra olvasd fel a válaszlehetőségeket!]

	Sokkal könnyebb	Inkább könnyebb	Körülbelül ugyanolyan	Inkább nehezebb	Sokkal nehezebb	NT/NV
a. …a gazdagok számára, mint a szegények számára?	1	2	3	4	5	9
b. …az idősek számára, mint a fiatalok számára?	1	2	3	4	5	9
c. …a nők számára, mint a férfiak számára?	1	2	3	4	5	9
d. …a magyar állampolgárok számára, mint a magyar állampolgársággal nem rendelkezők számára?	1	2	3	4	5	9

8. A súlyos egészségügyi problémáknak sokféle oka lehet. Mennyire ért egyet a következő állításokkal? Mennyire ért egyet azzal, hogy az emberek azért küzdenek súlyos egészségügyi problémákkal, mert…

[csak egy válasz soronként – KÉRDEZŐ: szükség esetén újra olvasd fel a válaszlehetőségeket!]

	Teljesen egyetért	Egyetért	Egyet is ért meg nem is	Nem ért egyet	Egyáltalán nem ért egyet	NT/NV
a. …olyan életmódot folytattak, ami károsította az egészségüket	1	2	3	4	5	9

b. …a munkahelyükön vagy a lakóhelyükön őket érő környezeti hatások miatt	1	2	3	4	5	9
c. …a genetikai adottságaik miatt	1	2	3	4	5	9
d. …mert szegények	1	2	3	4	5	9

9. Mennyire ért egyet a következő állítással: „Az alternatív gyógymódok/természetgyógyászat jobb megoldást kínálnak az egészségügyi problémákra, mint a hagyományos nyugati orvostudomány”

[csak egy válasz]

1 – Teljesen egyetért
2 – Egyetért
3 – Egyet is ért meg nem is
4 – Nem ért egyet
5 – Egyáltalán nem ért egyet

9 – NT X –

10. Mennyire ért egyet a következő állításokkal a magyar orvosokra nézve általában? Teljesen egyetért, egyetért, egyet is ért meg nem is, nem ért egyet, vagy egyáltalán nem ért egyet azzal, hogy…

[csak egy válasz soronként – KÉRDEZŐ: szükség esetén újra olvasd fel a válaszlehetőségeket!]

	Teljesen egyetért	Egyetért	Egyet is ért meg nem is	Nem ért egyet	Egyáltalán nem ért egyet	NT/NV
a. Mindent összevetve, az orvosokban meg lehet bízni	1	2	3	4	5	9
b. Az orvosok szaktudása nem olyan jó, mint kellene lennie	1	2	3	4	5	9
c. Az orvosokat jobban érdekli az, hogy mennyit keresnek, mint a betegeik	1	2	3	4	5	9

11. Az elmúlt 12 hónapban használta-e, és ha igen, milyen gyakran az internetet bármilyen eszközön (például számítógépen, tableten, vagy okostelefonon) arra, hogy egészségügyi vagy orvosi információkat keressen a saját maga vagy másvalaki számára?

[csak egy válasz]

1 – Naponta többször
2 – Egyszer egy nap
3 – Hetente többször
4 – Havonta többször
5 – Évente többször
6 – Soha, vagy szinte soha

9 – NT X –
77 – nincs internethozzáférése

12. Az elmúlt 12 hónapban használta-e, és ha igen, milyen gyakran az internetet arra, hogy a következő témákban információkat keressen?

[csak egy válasz soronként – KÉRDEZŐ: szükség esetén újra olvasd fel a válaszlehetőségeket!]

	Soha	Ritkán	Néha	Gyakran	Nagyon gyakran	NT/NV
a. Egészséges életmóddal kapcsolatos információk	1	2	3	4	5	9
b. Szorongással, stresszel, vagy hasonló problémákkal kapcsolatos információk	1	2	3	4	5	9
c. Védőoltásokkal kapcsolatos információk	1	2	3	4	5	9

13. Mennyire ért egyet a következő állításokkal? Az elmúlt 12 hónapban az interneten talált információk…

[csak egy válasz soronként – KÉRDEZŐ: szükség esetén újra olvasd fel a válaszlehetőségeket!]

	Teljesen egyetért	Egyetért	Egyet is ért meg nem is	Nem ért egyet	Egyáltalán nem ért egyet	NT/NV
a. ...segítségével egészségesebb életmódot tud folytatni	1	2	3	4	5	9
b. …segítettek megérteni valamit, amit egy orvos próbált elmagyarázni Önnek	1	2	3	4	5	9

14. Mennyire ért egyet a következő állításokkal?

[csak egy válasz soronként – KÉRDEZŐ: szükség esetén újra olvasd fel a válaszlehetőségeket!]

	Teljesen egyetért	Egyetért	Egyet is ért meg nem is	Nem ért egyet	Egyáltalán nem ért egyet	NT/NV
a. Az internet hasznos segítség annak eldöntésében, hogy a tünetek elég súlyosak-e ahhoz, hogy az ember orvoshoz forduljon.	1	2	3	4	5	9
b. Az internet hasznos annak ellenőrzésére, hogy az orvos megfelelő tanáccsal látja-e el az embert.	1	2	3	4	5	9
c. NEM könnyű megkülönböztetni az interneten a megbízható és a nem megbízható információkat az egészséggel kapcsolatosan.	1	2	3	4	5	9

15. Mennyire ért egyet következő, védőoltásokkal kapcsolatos állításokkal?

[csak egy válasz soronként – KÉRDEZŐ: szükség esetén újra olvasd fel a válaszlehetőségeket!]

	Teljesen egyetért	Egyetért	Egyet is ért meg nem is	Nem ért egyet	Egyáltalán nem ért egyet	NT/NV
a. Összességében az oltás többet árt, mint használ.	1	2	3	4	5	9
b. Jobb a természetes immunitás kialakítása az adott betegségen átesve, mint védőoltásban részesülni.	1	2	3	4	5	9

16. Az elmúlt 4 hétben milyen gyakran…

[csak egy válasz soronként – KÉRDEZŐ: szükség esetén újra olvasd fel a válaszlehetőségeket!]

	Soha	Ritkán	Néha	Gyakran	Nagyon gyakran	NT/NV
a. …esett nehezére a munkája vagy házimunka végzése egészségügyi problémák miatt?	1	2	3	4	5	9
b. …voltak testi fájdalmai?	1	2	3	4	5	9
c. …érezte magát boldogtalannak vagy lehangoltnak?	1	2	3	4	5	9
d. …érezte úgy, hogy elvesztette az önbizalmát?	1	2	3	4	5	9
e. …érezte úgy, hogy nem tud úrrá lenni a problémáin?	1	2	3	4	5	9

17.
Az elmúlt 12 hónapban milyen gyakran látogatott el, vagy látogatta meg Önt…

[csak egy válasz soronként – KÉRDEZŐ: szükség esetén újra olvasd fel a válaszlehetőségeket!]

	Soha	Ritkán	Néha	Gyakran	Nagyon gyakran	NT/NV
a. orvoshoz/orvos?	1	2	3	4	5	9
b. természetgyógyász/alternatív gyógyász/-hoz?	1	2	3	4	5	9

18. Az elmúlt 12 hónapban előfordult-e, hogy NEM kapott meg orvosi kezelést, amire szüksége volt, mert…

[csak egy válasz soronként – KÉRDEZŐ: szükség esetén újra olvasd fel a válaszlehetőségeket!]

	Igen	Nem	Nem volt szüksége orvosi kezelésre/nem releváns	NT/NV
a. …nem tudta megfizetni?	1	2	3	9
b. …nem tudott elszabadulni a munkahelyéről vagy más kötelezettség miatt?	1	2	3	9
c. …a várólista túl hosszú volt?	1	2	3	9

19. Mennyire valószínű, hogy ha súlyosan megbetegedne, megkapná a legjobb kezelést, ami Magyarországon elérhető?
[csak egy válasz]

1 – Biztos, hogy megkapná
2 – Valószínű, hogy megkapná
3 – Egyenlő eséllyel kapnám vagy nem kapná meg
4 – Valószínűleg nem kapná meg
5 – Biztos, hogy nem kapná meg

9 – NT X –

20. Általában véve mennyire elégedett vagy elégedetlen a magyar egészségügyi rendszerrel?

[csak egy válasz]

1 – Rendkívül elégedett
2 – Nagyon elégedett
3 – Eléggé elégedett
4 – Sem elégedett, sem elégedetlen
5 – Eléggé elégedetlen
6 – Nagyon elégedetlen
7 – Rendkívül elégedetlen
9 – NT X –

21. Mennyire volt elégedett a kapott kezeléssel, amikor…

[csak egy válasz soronként – KÉRDEZŐ: szükség esetén újra olvasd fel a válaszlehetőségeket!]

	Rendkívül elégedett	Nagyon elégedett	Eléggé elégedett	Sem elégedett, sem elégedetlen	Eléggé elégedetlen	Nagyon elégedetlen	Rendkívül elégedetlen	Nem releváns	NT/NV
a. …legutóbb orvosnál járt?	1	2	3	4	5	6	7	8	9
b. …amikor legutóbb természetgyógyásznál/alternatív gyógyásznál járt?	1	2	3	4	5	6	7	8	9

22. Dohányzik-e Ön - és ha igen, naponta körülbelül hány szál cigarettát szív el?

[csak egy válasz]

1 – Nem dohányzik, és sohasem dohányzott
2 – Nem dohányzik, de régebben dohányzott
3 – 1-5 szál cigarettát szív el egy nap
4 – 6-10 szál cigarettát szív el egy nap
5 – 11-20 szál cigarettát szív el egy nap
6 – 21-40 szál cigarettát szív el egy nap
7 – Több mint 40 szál cigarettát szív el egy nap
9 – NT X –

23. Ön milyen gyakran…

[csak egy válasz soronként – KÉRDEZŐ: szükség esetén újra olvasd fel a válaszlehetőségeket!]

	Soha	Havonta egyszer vagy ritkábban	Havi több alkalommal	Heti több alkalommal	Napi rendszerességgel	NT/NV
a. …fogyaszt 4 vagy több alkoholos italt egy nap?	1	2	3	4	5	9
b. …végez testmozgást legalább 20 percen keresztül, aminek során megizzad vagy a szokásosnál gyorsabban veszi a levegőt?	1	2	3	4	5	9
c. …fogyaszt friss gyümölcsöt vagy zöldséget?	1	2	3	4	5	9

24. Milyen az Ön egészsége általában?

5 – nagyon jó
4 – jó
3 – kielégítő
2 – rossz
1 – nagyon rossz
9 – NT X –

25. Van Önnek valamilyen hosszantartó betegsége, krónikus állapota, vagy fogyatékossága?

[csak egy válasz]

1 – Igen
2 – Nem
9 – NT X –

26. Mi az Ön…
a. magassága centiméterben: ________cm / NT/NV - 9
b. súlya kilogrammban: ________kg / NT/NV - 9

27. Ön hogy gondolja: a magyar kormánynak legyen, vagy ne legyen joga megtenni a következőket egy súlyos járvány idején?

[csak egy válasz soronként – KÉRDEZŐ: szükség esetén újra olvasd fel a válaszlehetőségeket!]

	Mindenképpen legyen joga	Inkább legyen joga	Inkább ne legyen joga	Semmiképpen se legyen joga	NT/NV
a. Üzletek és munkahelyek bezárása	1	2	3	4	9
b. Megkövetelni, hogy az emberek maradjanak otthon	1	2	3	4	9
c. Digitális (mobiltelefonos) megfigyelést használni a fertőzött emberek követésére	1	2	3	4	9
d. Megkövetelni, hogy ez emberek maszkot viseljenek	1	2	3	4	9
e. Betiltani a nyilvános rendezvényeket	1	2	3	4	9

REQUIRED ITEM WITH BACKGROUND QUESTIONS

<Note to ISSP members: Q28 is a compulsory background variable following a module-specific obligatory coding scheme. The question should be asked in a way (wording and categories) relevant to your country. However, please include the category "Have no health insurance" even if only a small number of your respondents are likely to place themselves in this category. The answers of each respondent should be coded into one of the following categories.>

28. Milyen típusú egészségbiztosítással rendelkezik Ön?

1 – Nincs egészségbiztosítása
2 – állami egészségbiztosítás (A)
3 – magán egészségbiztosítás (B)
4 – munkáltató/szakszervezet által nyújtott egészségbiztosítás (C)
5 – állami egészségbiztosítás és kiegészítő/magán biztosítás (A+B)
6 – állami egészségbiztosítás és munkáltató/szakszervezet által nyújtott egészségbiztosítás (A+C)
7 - munkáltató/szakszervezet által nyújtott egészségbiztosítás és kiegészítő/magán biztosítás (B+C)
8 - állami egészségbiztosítás, kiegészítő/magán biztosítás és munkáltató/szakszervezet által nyújtott egészségbiztosítás (A+B+C)
9 – Egyéb, éspedig: …………………..
99 – NT X –

OPCIONÁLIS ITEMEK

29. Ön hogy gondolja: a magyar kormánynak legyen, vagy ne legyen joga megtenni a következőket egy súlyos járvány idején?

[csak egy válasz soronként – KÉRDEZŐ: szükség esetén újra olvasd fel a válaszlehetőségeket!]

	Mindenképpen legyen joga	Inkább legyen joga	Inkább ne legyen joga	Semmiképpen se legyen joga	NT/NV
a. A betegséget bizonyítottan hordozó emberek elkülönítése	1	2	3	4	9

b. A kötelező oktatás felfüggesztése, és az iskolák és óvodák bezárása	1	2	3	4	9
c. Az országhatárok lezárása	1	2	3	4	9

30. Az, ahogy a Covid-19 járványt kezelték Magyarországon, inkább növelte, vagy csökkentette az Ön bizalmát…

[csak egy válasz soronként – KÉRDEZŐ: szükség esetén újra olvasd fel a válaszlehetőségeket!]

	Nagyban növelte	Kissé növelte	Se nem növelte, se nem csökkentette	Kissé csökkentette	Nagyban csökkentette	NT/NV
a. …az egészségügyi rendszerben?	1	2	3	4	5	9
b. …a kormányban?	1	2	3	4	5	9

31. A munkájára gondolva a Covid-19 járványt megelőzően és jelenleg, a következő állítások közül melyik jellemzi leginkább az Ön foglalkoztatási helyzetét:

[csak egy válasz]

1 – Nem volt munkám a járvány előtt, és most sincs
2 – Ugyanaz a munkahelyem, mint a járvány előtt volt
3 – Elvesztettem a munkám a járvány miatt, és most máshol dolgozom
4 – Elvesztettem a munkám a járvány miatt, és nem találtam új munkát
5 – Nem volt munkám a járvány előtt, de most van
6 – Munkahelyet váltottam vagy otthagytam a munkahelyem a járványtól független okokból
9 – NT X –

32. A Covid-19 járvány előtti időszakot összehasonlítva a jelenlegi helyzettel, mit mondana: nőtt, csökkent, vagy nagyjából ugyanakkora maradt a háztartása jövedelme?

[csak egy válasz]

1 – Sokat nőtt
2 – Kissé nőtt
3 – Nagyjából ugyanakkora maradt
4 – Kissé csökkent
5 – Sokat csökkent
9 – NT X –

33. Arra gondolva, hogy milyen gyakran találkozott személyesen a tágabb családjával és barátaival a Covid-19 járványt megelőzően, mit mondana: most gyakrabban, ritkábban, vagy körülbelül ugyanolyan gyakorisággal találkozik velük személyesen?

[csak egy válasz]

1 – Sokkal gyakrabban
2 – Kicsit gyakrabban
3 – Körülbelül ugyanolyan gyakorisággal
4 – Kicsit ritkábban

5 – Sokkal ritkábban
9 – NT X –

34. Különböző vélemények létezhetnek egyes egészségügyi problémákban szenvedő emberekről. Mennyire ért egyet a különböző állításokkal?

[csak egy válasz soronként – KÉRDEZŐ: szükség esetén újra olvasd fel a válaszlehetőségeket!]

	Teljesen egyetért	Egyetért	Egyet is ért meg nem is	Nem ért egyet	Egyáltalán nem ért egyet	NT/NV
a. A legtöbben azért lesznek erősen túlsúlyosak, mert lusták	1	2	3	4	5	9
b. A legtöbben, akiknek pozitív lett a Covid-19 tesztje, a gondatlanságuk miatt kapták el a vírust	1	2	3	4	5	9

MINDENKITŐL!

401. Élete folyamán hány gyermeke született Önnek?

.........gyermeke
0 – nem született még gyermeke
X –

402. Mennyi az Ön havi nettó, adózás utáni összes[1] jövedelme?

.... Ft/hó
8 – M 9 – NT →

X –

403.a. KÉRDEZŐ: fordítsd a válaszadó felé a laptopot/tabletet!
És azt megmondaná-e, hogy a közvetkező kategóriák közül, melyikbe tartozik?

01 – 20 ezer Ft, vagy kevesebb
02 – 21 - 40 ezer Ft
03 – 41 - 70 ezer Ft
04 – 71 - 100 ezer Ft
05 – 101 - 150 ezer Ft
06 – 151 - 200 ezer Ft
07 – 201 - 300 ezer Ft
08 – 301 - 500 ezer
09 – 500 ezer Ft felett
88 – M 99 – NT X –

403. HA NEM EGYEDÜL ÉL

Mindent egybevetve, mennyi az Önök háztartásának nettó (adózás utáni) havi összjövedelme (beleértve az Ön jövedelmét is)?

.... Ft/hó
8 – M 9 – NT →

X –

404.a. KÉRDEZŐ: fordítsd a válaszadó felé a laptopot/tabletet!
És azt megmondaná-e, hogy a következő kategóriák közül, melyikbe tartozik?

01 – 60 ezer Ft, vagy kevesebb
02 – 61 - 90 ezer Ft
03 – 91 - 120 ezer Ft
04 – 121 - 150 ezer Ft
05 – 151 - 200 ezer Ft
06 – 201 - 300 ezer Ft
07 – 301 - 500 ezer Ft
08 – 501 ezer -1 millió Ft
09 – 1 millió Ft felett
88 – M 99 – NT X –

MINDENKITŐL!

404. Előfordult az elmúlt évben, hogy pénzhiány miatt nem tudták befizetni a...

		Igen	Nem	NT	Nincs ilyen költségük	
a.	Közüzemi díjat?	1	2	9	0	X
b.	Lakáshitel-törlesztést?	1	2	9	0	X
c.	Egyéb hitel törlesztést	1	2	9	0	X

[1] Ha több forrásból van jövedelme, összesen és átlagosan mennyi?

405. Hogy érzi, Önök anyagilag...

5 – gondok nélkül élnek
4 – beosztással jól kijönnek
3 – éppen, hogy kijönnek a havi jövedelmükből
2 – hónapról-hónapra anyagi gondjaik vannak, vagy
1 – nélkülözések között élnek?

9 – NT X –

406. Magyarországon vannak magasabb és alacsonyabb társadalmi helyzetű emberek.
Képzeljen el egy tíz fokozatú létrát, aminek legfelső foka a legmagasabb társadalmi helyzetű embereket, legalsó foka pedig a legalacsonyabb helyzetűeket jelenti. Most hol helyezné el ezen a létrán Önmagát?

lent 01 02 03 04 05 06 07 08 09 10 fent

99 – NT X –

407. Tagja-e Ön valamelyik szakszervezetnek?

1 – igen
2 – nem → **408.a.** Régebben tag volt-e?
9 – NT → 1 – igen 2 – nem 9 – NT X -
X –

408. Részt vett-e Ön a legutóbbi, 2018. áprilisi parlamenti választáson?

1 – igen → dx409a
2 – nem
0 – nem szavazhatott
8 – VM
9 – NEM EMLÉKSZIK X –

409A.

Kérem, mondja meg, hogy Ön a parlamenti választáson melyik párt listájára adta le szavazatát?
KÉRDEZŐ: fordítsd a válaszadó felé a laptopot/tabletet!

01 – FIDESZ-KDNP
02 – MSZP, Magyar Szocialista Párt
03 – Jobbik Magyarországért
04 – LMP, Lehet Más a Politika
05 – Együtt-a Korszakváltók Pártja
06 – Demokratikus Koalíció
07 – PM, Párbeszéd Magyarországért
08 – Momentum
10 – egyéb párt, éspedig: …………

88 – M 99 – NT X –

MINDENKITŐL!

409. Hány szobás ez a lakás?

FÉLSZOBA IS SZOBÁNAK SZÁMÍT!

..................szobás
X –

410. Hány négyzetméter a lakás alapterülete?

.................négyzetméter
999 – NT X –

411. Önök tulajdonosként laknak ebben a lakásban / házban?

1 – igen 2 – nem 9 – NT X –

412. **HA TULAJDONOSKÉNT LAKNAK**: Mennyit ér ez a lakás / ház?
HA NEM TULAJDONOSKÉNT LAKNAK: Mennyit érne ez a lakás/ház, ha az Ön tulajdona lenne?

.... Ft
8 – M 9 – NT X –

413. Van Önnek / Önöknek...

	van	nincs	NT	
a. bankkártyájuk vagy hitelkártyájuk?	1	2	9	X
b. részvényük?	1	2	9	X
c. lakossági folyószámlájuk?	1	2	9	X
d. önkéntes kiegészítő nyugdíjbiztosításuk?	1	2	9	X
e. életbiztosításuk?	1	2	9	X

414. Van Önöknek...

	van	nincs	
a. Automata mosógépük?	1	2	X
b. Vezetékes telefonjuk?	1	2	X
c. Plazma vagy LCD TV-jük?	1	2	X
d. DVD-lejátszójuk?	1	2	X
e. Mosogatógépük?	1	2	X
f. Klímaberendezésük?	1	2	X
g. Személygépkocsijuk (saját vagy céges, amit magáncélra használhatnak)? **HA VAN:** Hány darab?	**db**	0	X
h. Számítógépük, laptop, tablet? **HA VAN:** Hány darab?	**db**	0	X
i. HA NINCS SZÁMÍTÓGÉP = X! Internet hozzáférésük?	1	2	X
j. Más lakóingatlanuk vagy nyaralójuk?	1	2	X

415. Van Önnek saját…

	van	nincs	
a. E-mail elérhetősége?	1	2	X
b. Facebook vagy hasonló (közösségi) oldala?	1	2	X

416. Ha egyszerűen és könnyen válthatna szolgáltatót, váltana Ön…

	Igen	Nem	Nem vesz igénybe ilyen szolgáltatást	NT	
a. áramszolgáltatót?	1	2	3	9	X
b. gázszolgáltatót?	1	2	3	9	X
c. vezetékes telefon szolgáltatót?	1	2	3	9	X
d. mobil telefon szolgáltatót?	1	2	3	9	X
e. kötelező gépjármű felelősségbiztosítást?	1	2	3	9	X

HA K13=1.

417. Milyen gyakran szokott Ön internetezni?

1 – naponta, naponta többször
2 – hetente többször
3 – hetente egyszer
4 – havonta többször
5 – havonta egyszer, vagy ritkábban
9 – NT X –

MINDENKITŐL!

418. Ön személy szerint használ-e mobiltelefont?

1 – igen 2 – nem 9 – NT X –

419. Milyen gyakran jár Ön misére, istentiszteletre, gyülekezetbe?

8 – soha
7 – ritkábban, mint évente
6 – évente egy-kétszer
5 – évente néhányszor
4 – kb. havonta egyszer
3 – havonta kétszer-háromszor
2 – hetente egyszer
1 – hetente többször
9 – NEM TUDJA

420. Ön milyen vallású?

protestáns: 01 – református (Kálvinista)

02 – evangélikus (Lutheránus)

03 – unitárius

04 – baptista

	05 –	metodista
	07 –	egyéb protestáns, éspedig: ....
katolikus:	08 –	római katolikus
	09 –	görögkatolikus
zsidó	10 –	zsidó
ortodox:	11 –	orosz ortodox
	12 –	görögkeleti
muzulmán	13 –	muzulmán
egyéb	14 –	egyéb nem keresztény: ....
	15 –	egyéb keresztény: ....

77 – nem tartozik felekezethez

99 – NEM TUDJA

X –

421. Ön melyik állítással tudná magát a legjobban jellemezni?

3 – Vallásos vagyok, az egyház tanításait követem. 0 – Nem vagyok vallásos.
2 – Vallásos vagyok a magam módján. 8 – MEGTAGADJA A VÁLASZT
1 – Nem tudom megmondani, hogy vallásos vagyok-e vagy sem. X –

422. Országunkban az emberek sokféle nemzetiséghez vagy etnikumhoz tartoznak. Ön milyen nemzetiségűnek tartja magát elsősorban?

01 — magyar
02 — német
03 — szerb
04 — horvát
05 — román
06 — szlovák
07 — roma / cigány
08 — egyéb, éspedig:…………………….
88 — MEGTAGADJA A VÁLASZT
99 — NEM TUDJA
X —

423. És milyen nemzetiségűnek tartja magát másodsorban?

00 — nem tartozik más nemzetiséghez
01 — magyar
02 — német
03 — szerb
04 — horvát
05 — román
06 — szlovák
07 — roma / cigány
08 — egyéb, éspedig:…………………….
88 — MEGTAGADJA A VÁLASZT
99 — NEM TUDJA
X —

MINDENKITŐL!

424. Köszönjük válaszait! Szeretném megkérdezni, hogy máskor is válaszolna-e kérdőívünkre?

1 – igen   2 – nem   X –

425. És telefonon válaszolna-e egy-egy rövid kérdőívre?

1 – igen   2 – nem   X –

426. És interneten keresztül válaszolna-e egy-egy rövid kérdőívre?

1 – igen   2 – nem   X –

428.– 430. HA VÁLASZOLNA SZEMÉLYESEN,TELEFONON ÉS/VAGY INTERNETEN

427. Kérem, írja alá ezt a lapot! CÍMKÁRTYA igazoljuk, hogy Ön hozzájárult nevének és címének megőrzéséhez azért, hogy máskor is meg tudjuk keresni Önt. Természetesen a címét és nevét nem adjuk ki más cégeknek.

405 – aláírta a címkártyát   2 – nem írta alá a címkártyát   X –

428. Kérem, adja meg, ha van, az e-mail címét.

1 – megadott e-mail címet
2 – nem adott meg e-mail címet
X –

ÍRD A CÍMKÁRTYÁRA!

429. Kérem, adja meg, ha van a mobiltelefonja számát és a vezetékes telefonja számát is (körzetszám és a telefonszám)!

ÍRD A CÍMKÁRTYÁRA!

430.a.) 1 – megadott mobiltelefonszámot
2 – nem adott meg
X –

430.b.) 1 – megadott vezetékes telefonszámot
2 – nem adott meg
X –

Még egyszer köszönjük, hogy válaszaival segítette munkánkat!

KÉRDEZÉS VÉGE: .... óra .... perc

KÉRDÉSEK A KÉRDEZŐNEK

I. Milyen jellegű épületben van a lakás?

1 – lakótelepi épület
2 – többlakásos zöldövezeti
3 – többlakásos nem zöldövezeti
4 – egyszintes iker- vagy családi ház
5 – többszintes iker- vagy családi ház
6 – hagyományos építésű parasztház
7 – tanya
8 – egyéb lakott épület
9 – nem tudod eldönteni
X –

II. A ház helye a településen belül:

1 – olcsó lakáspiaci övezet
2 – közepes, átlagos lakáspiaci övezet
3 – helyileg magasabbra értékelt lakáspiaci övezet
4 – helyileg a legmagasabbra értékelt lakáspiaci övezet
9 – nem tudod eldönteni
X –

III. A kérdezett szerinted roma származású?

1 – igen
2 – nem
X –

IV. Szerinted mennyit ér az a lakás / ház, amiben a kérdezett lakik?

....Ft-ot
9 – nem tudod eldönteni
X –

Köszönjük munkádat!
